# Effects of Laser *In Situ* Keratomileusis and Small-Incision Lenticule Extraction on Corneal Biomechanical Behavior: A Finite Element Analysis

**DOI:** 10.3389/fbioe.2022.855367

**Published:** 2022-04-11

**Authors:** Chenyan Wang, Xiaona Li, Yuan Guo, Rui He, Hongmei Guo, Weiyi Chen

**Affiliations:** ^1^ College of Biomedical Engineering, Taiyuan University of Technology, Taiyuan, China; ^2^ Department of Excimer Laser, Shanxi Eye Hospital, Taiyuan, China

**Keywords:** finite element analysis, biomechanics, laser *in situ* keratomileusis, small-incision lenticule extraction, cornea

## Abstract

Myopia, which is the result of the uncoordinated development of the eyeball, has become a major public health focus worldwide. Laser *in situ* keratomileusis (LASIK) and small-incision lenticule extraction (SMILE) have been successfully used in modern corneal refractive surgery. However, there are still controversies about postoperative results of LASIK and SMILE. In this study, a three-dimensional finite element model of the cornea was constructed based on the elevation and pachymetry data of a female volunteer. Surgical parameters, magnitudes of myopic correction, and intraocular pressure (IOP) were varied. Furthermore, an iterative algorithm was applied to retrieve the free-stress state of the intact corneal model, LASIK model, and SMILE model. To better evaluate the differences between LASIK and SMILE procedures, the displacement and Von Mises stress on the anterior and posterior corneal surface along the *x-* and *y*-axes were analyzed. Results for the zero-pressure model showed larger displacement compared to the image-based corneal model, suggesting that the initial corneal pre-stress stiffens the response of the cornea, both in the intact cornea and under refractive surgery. In addition, the displacement on the corneal surface in LASIK (both zero-pressure and image-based model) was obviously higher than that of the SMILE model. In contrast, SMILE increased Von Mises stress in the corneal cap and reduced Von Mises stress in the residual stromal bed compared with the LASIK model. However, the maximum Von Mises stress in the SMILE model was still smaller than that of the LASIK model. Moreover, the displacement and Von Mises stress on the residual stromal bed increased linearly with IOP. Overall, LASIK and SMILE refractive surgery could change biomechanical behaviors of the cornea. Compared to LASIK refractive surgery, SMILE may present a lower risk of ectasia. Creating a corneal cap rather than a corneal flap may have an advantage in improving corneal biomechanical stability.

## Introduction

Myopia has become a major global public health focus (Li et al., 2021). The proportion of people with myopia is gradually increasing, especially in East Asian regions where the incidence of young adults is approximately 80%–90% (Morgan et al., 2018). Furthermore, it has been reported that the myopia prevalence rate can be as high as 49.8% of the world population (about 4.8 billion people) by the year 2050 (Holden et al., 2016). In order to improve the quality of life, there are growing demands for restoring normal visual function. Corneal refractive surgery was introduced to achieve permanent refractive correction and reduce dependence on glasses and contact lenses (Simonini and Pandolfi, 2015; Bao et al., 2018).

Laser *in situ* keratomileusis (LASIK) and small-incision lenticule extraction (SMILE) have been successfully used in modern corneal refractive surgery with good postoperative results. Since 2003, the femtosecond laser had been introduced to replace the mechanical microkeratome in the creation of corneal flap during the LASIK surgery (Nordan et al., 2003). The combination of femtosecond lasers and high-resolution laser instruments makes LASIK an effective method for achieving better control of the flap depth and ablation profile (Roberts, 2000; Farjo et al., 2013). LASIK has been performed to correct myopia with a satisfaction rate of over 95% (Roy et al., 2014). SMILE surgery is a newly developed technique that removes the refractive lenticule through a small incision with less damage to the corneal microstructure (Sekundo et al., 2011; Zhang et al., 2015). Some researchers (Ang et al., 2013; Ivarsen et al., 2014; Shen et al., 2016) suggested that the SMILE approach could provide excellent clinical outcomes in regard to efficacy, predictability, and safety.

In general, the biomechanical behavior of the cornea is related to geometry shapes (Ariza-Gracia et al., 2015), such as corneal central thickness, curvature, and tomography. It is inevitable that lenticule removal and corneal flap/cap creation will directly lead to corneal geometry changes. Theoretically, the absence of a corneal flap creation was believed to allow SMILE procedure benefits beyond those of LASIK because of greater corneal biomechanical strength. Wu et al. investigated the corneal higher-order aberrations after SMILE and FS-LASIK through a comparative experiment (Wu and Wang, 2016). They found that SMILE could decrease corneal spherical aberration compared with FS-LASIK surgery. However, there was little difference in postoperative clinical results between LASIK and SMILE (Vestergaard et al., 2013; Lin et al., 2014; Moshirfar et al., 2015). Agca et al. (2014) conducted a prospective comparative case series and found no difference between SMILE and FS-LASIK in postoperative corneal hysteresis and corneal resistance factor (Agca et al., 2014). Therefore, there is a clear need for additional research to investigate whether the SMILE procedure has advantages over LASIK on postoperative outcomes.

The finite element method is not only a useful tool for simulating the biomechanical properties of the cornea, but also plays an important role in the design of clinical refractive surgery and the prediction of postoperative complications such as keratoconus. In the last 2 decades, the mechanical behavior of the human cornea under normal conditions or refractive surgery has been discussed by methods of numerical simulation (Pandolfi and Manganiello, 2006; Roy and Dupps, 2009; Nguyen and Boyce, 2011; Ariza-Gracia et al., 2015; Katzengold et al., 2021). Katzengold et al. simulated the LASIK surgery and investigated its biomechanical consequences through the finite element method (Katzengold et al., 2021). Roy et al. constructed a human whole-eye finite element model to investigate the impact of corneal properties on biomechanical responses before and after the LASIK surgery (Roy and Dupps, 2009). However, only a few studies had compared the displacement distributions and stress between the LASIK and SMILE procedures using a three-dimensional finite element model.

The purpose of this study was to evaluate differences in displacement and stress on the treated residual stromal bed and corneal flap/cap of the postoperative state resulting from the LASIK and SMILE model, and taking into account the free-stress configuration of the corneal. Furthermore, this study also analyzed biomechanical behaviors of the cornea under different ablation depths and loading conditions. These simulation results could help to clarify whether SMILE is superior to LASIK from a biomechanical view, and provide a valuable reference for the ophthalmologist.

## Materials and Methods

### Geometrical Model of the Cornea

**FIGURE 1:**
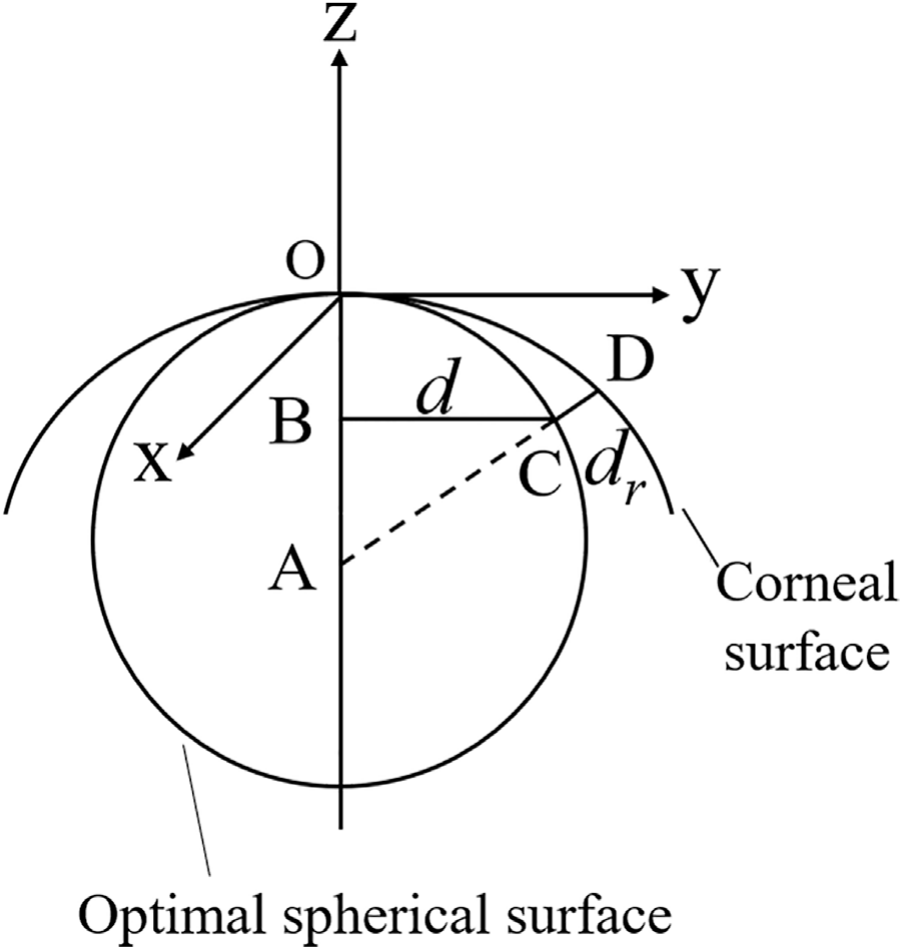
Diagram for the coordinates of the anterior corneal surface.

Clinical data from a healthy female volunteer was collected by the Oculus Pentacam. The Pentacam provides the coordinates (*x*, *y*) of points belonging to the anterior corneal surface, the pachymetry data for each point, the optimal spherical surface, and the distance from the point on the anterior corneal surface to the optimal spherical surface. Therefore, the value of each point in the *z*-axis direction can be calculated as (Figure 1)

$$d={\left(x^{2}+y^{2}\right)}^{1/2}$$ (1)

$$z=-\left(r-{\left({\left(r+d_{r}\right)}^{2}-d^{2}\right)}^{1/2}\right)$$ (2)

Where the optimal sphere is a circle centered at A (0, 0, -r). *d* is the distance from the corneal surface to the *z*-axis. *d*_*r*_ is the distance from the corneal surface to the optimal sphere surface.

**FIGURE 2:**
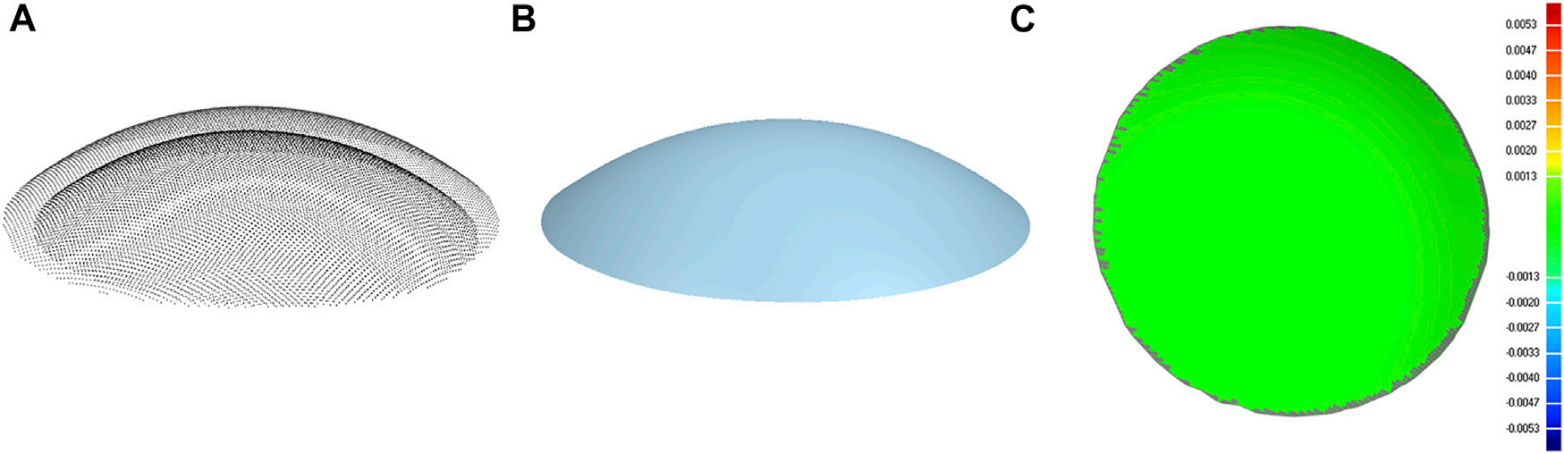
Corneal surface reconstruction. **(A)** Cloud of points of the anterior and posterior corneal surface. **(B)** Geometrical model of the cornea. **(C)** Contour map of the error between the point cloud data and corneal geometrical model (both anterior and posterior corneal surface).

The points on the posterior corneal surface are obtained by point-to-point subtraction of the anterior surface and the pachymetry data. There were 9,101 and 7,319 coordinate points on the anterior and posterior corneal surface, respectively, and were stored in.csv files. The coordinate points were then imported into the Geomagic studio 2015 (Raindrop, America), and a three-dimensional model of the cornea was reconstructed (Figure 2). The subject had given consent for the scientific use of the corneal morphology data. Moreover, ethical approval was obtained from the Ethics Committee of Taiyuan University of Technology (TYUT202107001).

### Refractive Procedure Simulation

To treat myopia, refractive surgery ablates a small lenticule from the corneal tissue. After surgery, the change of diopter can be expressed as (Munnerlyn et al., 1988)

$$S=\left(n-1\right)\left(\frac{1}{R_{2}}-\frac{1}{R_{1}}\right)$$ (3)

Where *S* is the surgical correction of myopia diopter. *R*_*1*_ and *R*_*2*_ are the initial and final radius of the corneal anterior surface. *n* = 1.377 is the refractive index of the cornea.

The shape of the lenticule is thick in the middle and thin around the edge. The thickness of the removed corneal tissue can be described according to the Munnerlyn equation, which is defined as

$$f\left(\text{r}\right)=\sqrt{R_{1}^{2}-r^{2}}-\sqrt{R_{1}^{2}-\frac{t^{2}}{4}}-\sqrt{{\left(\frac{R_{1}\left(n-1\right)}{n-1+R_{1}S}\right)}^{2}-r^{2}}+\sqrt{{\left(\frac{R_{1}\left(n-1\right)}{n-1+R_{1}S}\right)}^{2}-\frac{t^{2}}{4}}$$ (4)

Where *r* is the distance from a point to the optical axis, *t* is the diameter of the optical zone.

The maximum removal of corneal tissue is given by

$$f_{\max}=R_{1}-\sqrt{R_{1}^{2}-\frac{t^{2}}{4}}-\sqrt{{\left(\frac{R_{1}\left(n-1\right)}{n-1+R_{1}S}\right)}^{2}}+\sqrt{{\left(\frac{R_{1}\left(n-1\right)}{n-1+R_{1}S}\right)}^{2}-\frac{t^{2}}{4}}$$ (5)

**FIGURE 3:**
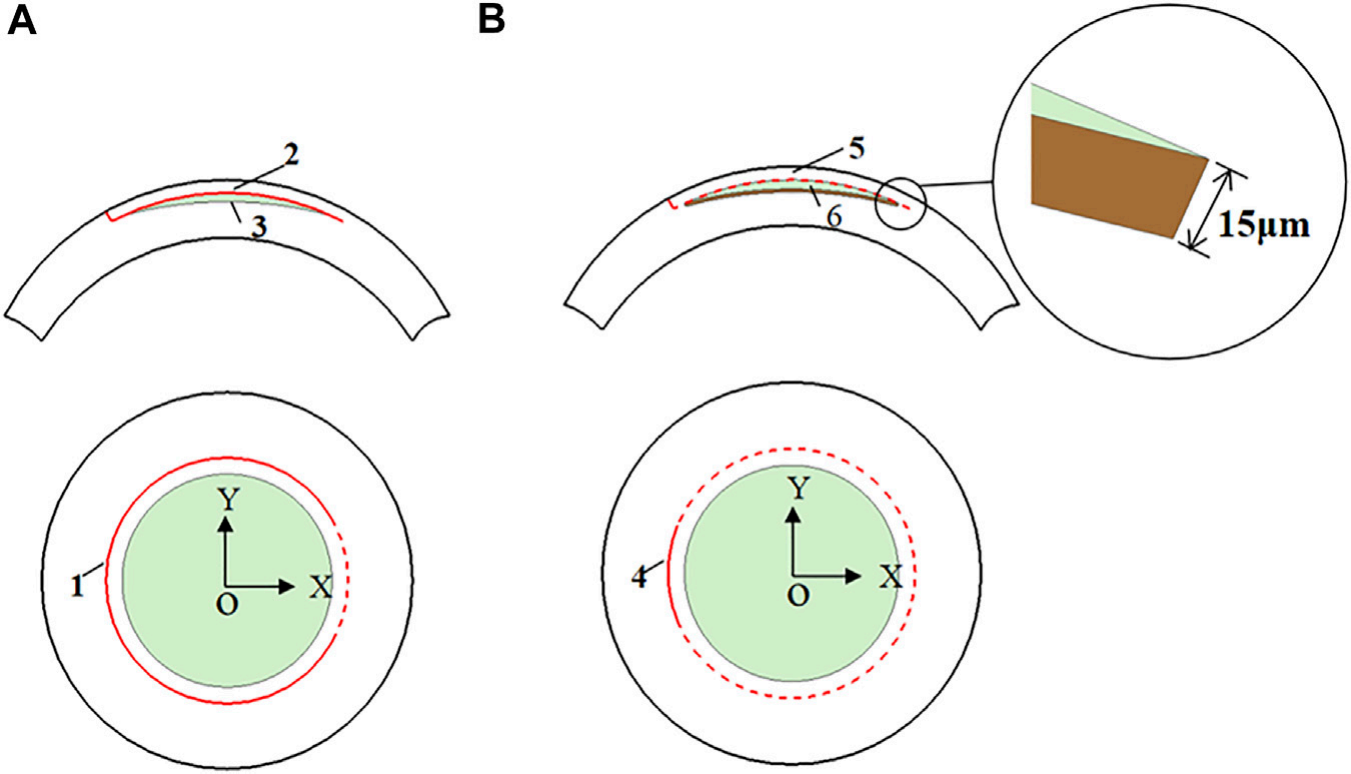
The model features of the LASIK and SMILE procedure. **(A)** LASIK procedure (1 flap opening incision; 2 corneal flap; 3 lenticule); **(B)** SMILE procedure (4 cap opening incision; 5 corneal cap; 6 lenticule).

In this study, the anterior and posterior corneal surfaces were reconstructed in Geomagic studio. The corneal surfaces were then imported into the Unigraphics NX (Siemens PLM, America) to create the LASIK and SMILE models. Furthermore, each model was assumed to have a single side-cut incision. For the LASIK model, the diameter of the corneal flap was 7.5 mm, and the thickness of the flap was set to be 120 μm. In order to shorten the differences between the two refractive approaches, the cap diameter of the SMILE model was 7.5 mm, and the thickness was also 120 μm (Jin et al., 2018). In addition, for the SMILE surgery, it also considered the actual ablation profile, i.e., an additional 15 μm of the lenticule for easy extraction (Figure 3). Moreover, the lenticule diameter (optical zone) was 6.5 mm for both two models. There were two scenarios for myopic corrections of -3D and -6D. The ablation depths are listed in Table 1.

**TABLE 1:** Ablation depths in the LASIK and SMILE model.

Myopic correction	LASIK ablation depth (mm)	SMILE ablation depth (mm)
**-3D**	42.25	57.25
**-6D**	84.5	99.5

### Zero-Pressure Configuration of the Cornea

**FIGURE 4:**
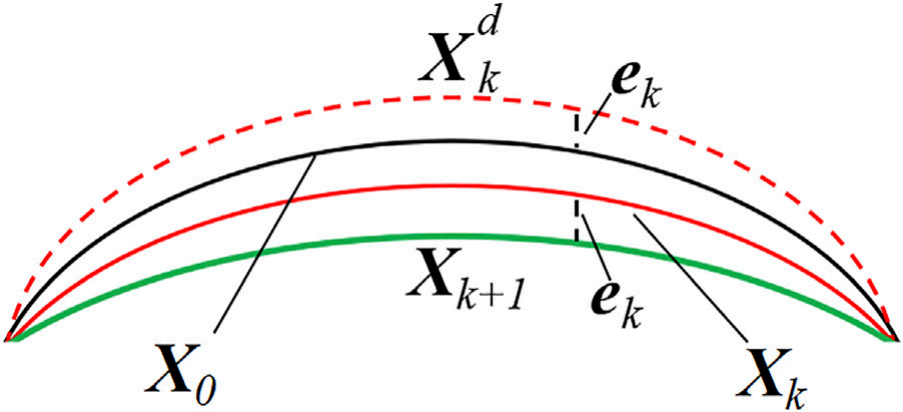
Zero-pressure iterative algorithm.

The geometry of the eye, measured *in vivo*, is in a state of deformation due to the presence of intraocular pressure (IOP). Thus, an accurate finite element analysis should recover the zero-pressure configuration of the cornea, i.e., the initial state in which the tissue is free from the IOP. Therefore, an iterative procedure is applied to find the zero-pressure geometry of the cornea (Figure 4).

In this study, the algorithm works iteratively by updating the nodal coordinates and comparing the values obtained from the clinical measurements with those provided by the finite element analysis.

$$X_{k+1}=X_{k}+X_{0}-X_{k}^{d}$$ (6)

Where ***X*** denotes an N×3 matrix storing the nodal coordinates of the corneal model, and N is the number of nodes in the finite element mesh. ***X***_0_ represents the geometrical coordinates of the corneal from the clinical data. ***X***_*k*_ is the nodal coordinates at the *k*th iteration. Then, a static finite element analysis was performed by applying physiological IOP to the posterior corneal surface. $X_{k}^{d}$ stands for an N×3 matrix that stores the deformation coordinate of each finite element mesh node under the IOP pressure. In the iterative algorithm, the nodal coordinates were set by the inp file in ABAQUS (Simulia, America).

$$e_{k}={\left\|X_{k}^{d}-X_{0}\right\|}_{\infty }$$ (7)

$$\frac{e_{k}}{\mathrm{CCT}}<\varepsilon$$ (8)

Where *e*_*k*_ is the infinite norm of the difference in nodal coordinates between $X_{k}^{d}$ and ***X***_0_. *ε* represents the relative tolerance error. CCT is the central corneal thickness, which in the present study was 515 μm. The iterative procedure continues until *ε* is less than a preset value. In this study, the allowable relative tolerance error is smaller than 0.25 μm (less than 0.05% of the CCT).

### Material Properties and Boundary Condition

The anisotropic hyperelastic material property was incorporated into the model to provide a more realistic representation of the cornea (Holzapfel et al., 2000). This property was modeled using Gasser–Holzapfel–Ogden’s (G-H-O) constitutive equation. For this study, the same mechanical properties and dispersion parameters were considered for the LASIK and SMILE models. The parameters of C_10_, D, k_1_, k_2_, and κ were 0.05 MPa, 0 MPa^−1^, 130.9 MPa, 2,490, and 0.33329, respectively (Ariza-Gracia et al., 2016).

For boundary conditions, the rim of the cornea was constrained in all degrees of freedom. The friction coefficient was 0.8 between the posterior flap/cap surface and the anterior stromal surface since this value could simulate the clinical situation (Katzengold et al., 2021). Loading was associated with intraocular pressure (IOP). Each model was solved using the IOP: 15 mmHg, 19 mmHg, 23 mmHg, 27 mmHg, and 31 mmHg. The finite element simulations were performed using ABAQUS.

As shown in Figure 3, the origin of the coordinate system was set at the center of the cornea. In order to compare different refractive surgeries, four paths were investigated, namely, the paths along the *x-* and *y*-axes on the anterior corneal surface, and the paths along the *x-* and *y*-axes on the posterior corneal surface. Moreover, displacements and Von Mises stresses along different paths were calculated.

## Results

### Displacement on the Corneal Surface

**FIGURE 5:**
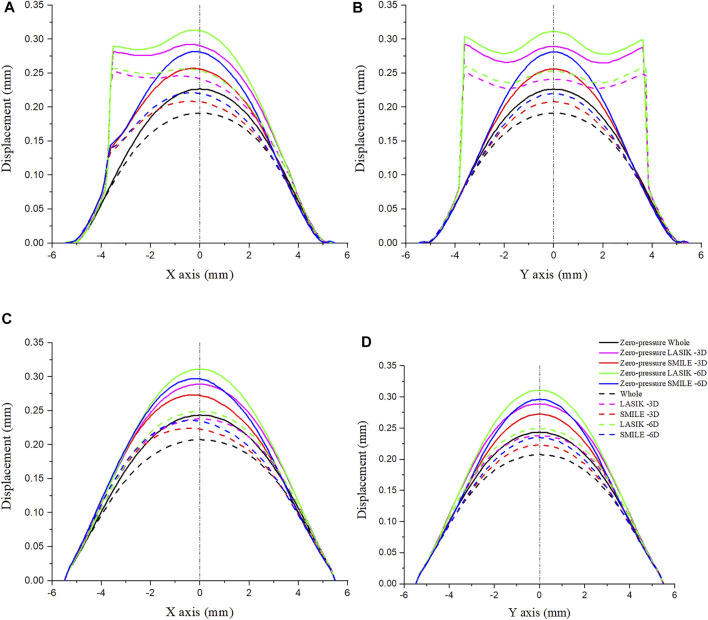
Comparison of displacement on the cornea along the different paths. The IOP was constant at 19 mmHg. **(A)** Displacement on the anterior corneal surface along the *x*-axis. **(B)** Displacement on the anterior corneal surface along the *y*-axis. **(C)** Displacement on the posterior corneal surface along the *x*-axis. **(D)** Displacement on the posterior corneal surface along the *y*-axis. (IOP = intraocular pressure; LASIK = laser *in situ* keratomileusis; SMILE = small-incision lenticule extraction).

Figure 5 shows the displacement along four paths for the intact cornea as well as for two different myopic refractive surgeries (LASIK and SMILE), which were obtained using the zero-pressure model and the image-based model. It was obvious that displacement variations along the same path on the anterior corneal surface were different between LASIK and SMILE. However, the trends of displacement along the same path were similar on the posterior corneal surface. In addition, incorporating the zero pressure of the cornea results in a softer corneal response to the IOP (higher displacement along the same path), as shown in Figure 5, where the zero-pressure configuration produces a shift in the displacement vs. path curve. For example, the maximum displacement on the anterior corneal surface was 0.221 and 0.188 mm for the zero-pressure and image-based intact corneal model.

To show a more comprehensive mechanical performance between different refractive surgeries, the myopic correction was set to -3D and -6D. Under the same refractive surgery, the more ablation depth is, the greater were displacements induced by the applied IOP. Moreover, the displacement along the *x-* and *y*-axes was obviously increased during the LASIK surgery, especially in the corneal flap. For the zero-pressure LASIK model with -6D refractive surgery, the corneal vertex displacement on the anterior surface was 0.034 mm larger than that of the SMILE model.

### Von Mises Stress on the Corneal Surface

**FIGURE 6:**
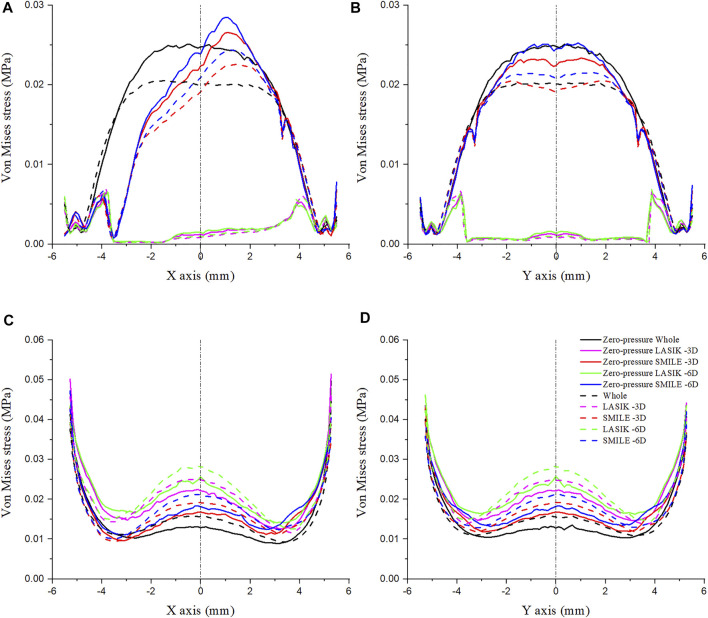
Comparison of Von Mises stress on the cornea along the different paths. The IOP was constant at 19 mmHg. **(A)** Von Mises stress on the anterior corneal surface along the *x*-axis. **(B)** Von Mises stress on the anterior corneal surface along the *y*-axis. **(C)** Von Mises stress on the posterior corneal surface along the *x*-axis. **(D)** Von Mises stress on the posterior corneal surface along the *y*-axis.

The Von Mises stress along the *x-* and *y*-axes was investigated for LASIK and SMILE (myopic correction with -3D and -6D) in zero- and non-zero-pressure states, as shown in Figure 6. IOP was constant at 19 mmHg. The maximum Von Mises stress of LASIK was higher than that of SMILE. In addition, for the same refractive surgery, the vertex stress was higher at the anterior corneal surface in the zero-pressure model compared to the image-based model, while the opposite was observed for the posterior corneal surface.

The Von Mises stress along the *x-* and *y*-axes of the anterior corneal surface showed obvious differences (Figures 6A,B). The stress along the *y*-axes tended to be bilaterally symmetric, with the maximum stress almost at the center of the cornea; however, the stress along the *x*-axis was not bilaterally symmetric, which may be due to the position of the corneal flap/cap incision. Furthermore, the Von Mises stress along the same path on the posterior corneal surface in the LASIK model was obviously higher than that of the SMILE model (Figures 6C,D). The results also showed that the stress distribution on the posterior corneal surface was nearly symmetrical. Moreover, a comparison of these plots showed that the stress increased with the myopic refractive correction.

### Effects of IOP on the Displacement and Von Mises Stress of the Cornea

**FIGURE 7:**
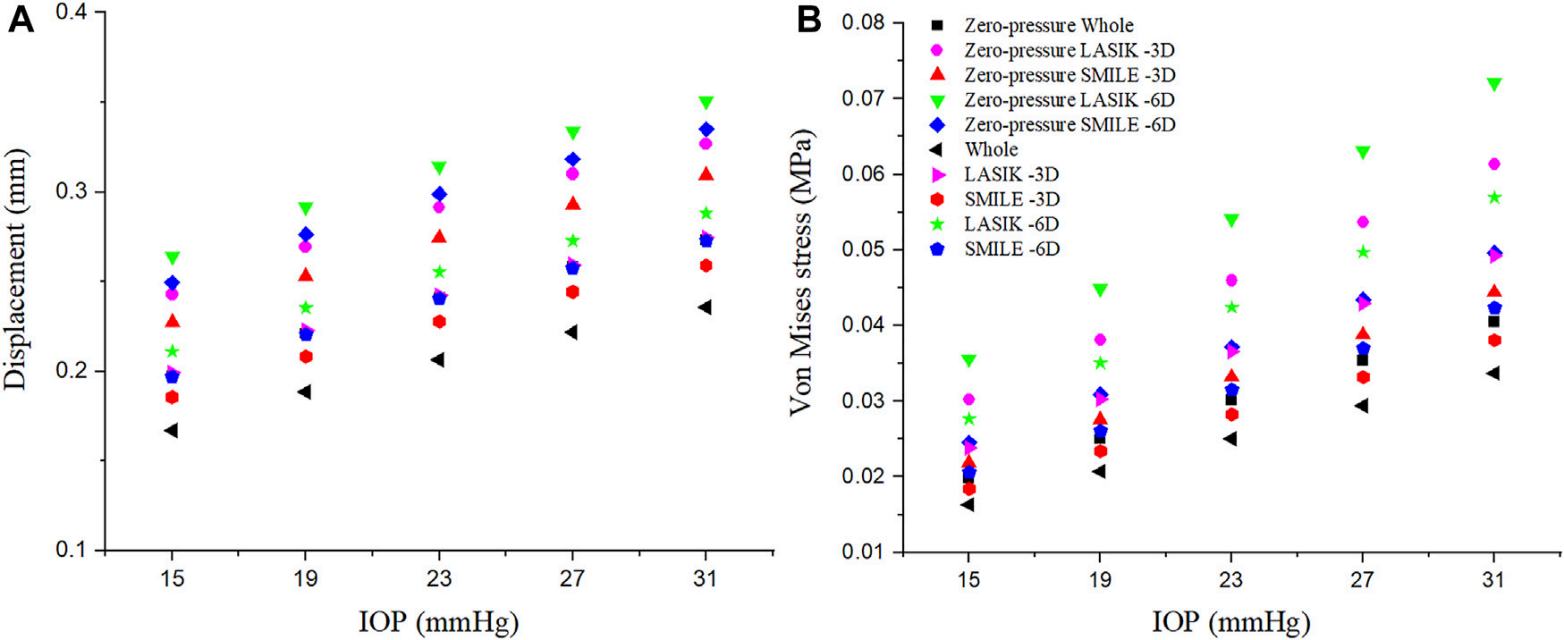
Effects of the IOP on the displacement and Von Mises stress of the corneal residual stromal bed vertex. The IOP was set at 15 mmHg, 19 mmHg, 23 mmHg, 27 mmHg, and 31 mmHg, respectively. **(A)** Displacement. **(B)** Von Mises stress.

The relationship between the displacement on the corneal residual stromal bed vertex and IOP is presented in Figure 7A. IOP was set to 15 mmHg, 19 mmHg, 23 mmHg, 27 mmHg, and 31 mmHg, respectively. There was an approximately linear relationship between corneal residual stromal bed vertex and IOP. In addition, under the same myopic correction, the displacement was greater in LASIK compared with SMILE (both zero-pressure and image-based model).

Similarly, the linear relationship between the Von Mises stress on the corneal residual stromal bed vertex and IOP could be seen in Figure 7B. When the IOP was constant, the Von Mises stress on the corneal residual stromal bed vertex of SMILE was smaller than that of LASIK (both zero-pressure and image-based model). Furthermore, it was obvious that considering the zero-pressure state of the cornea may lead to more deformation and stress.

### Displacement Distributions on the Cornea

**FIGURE 8:**
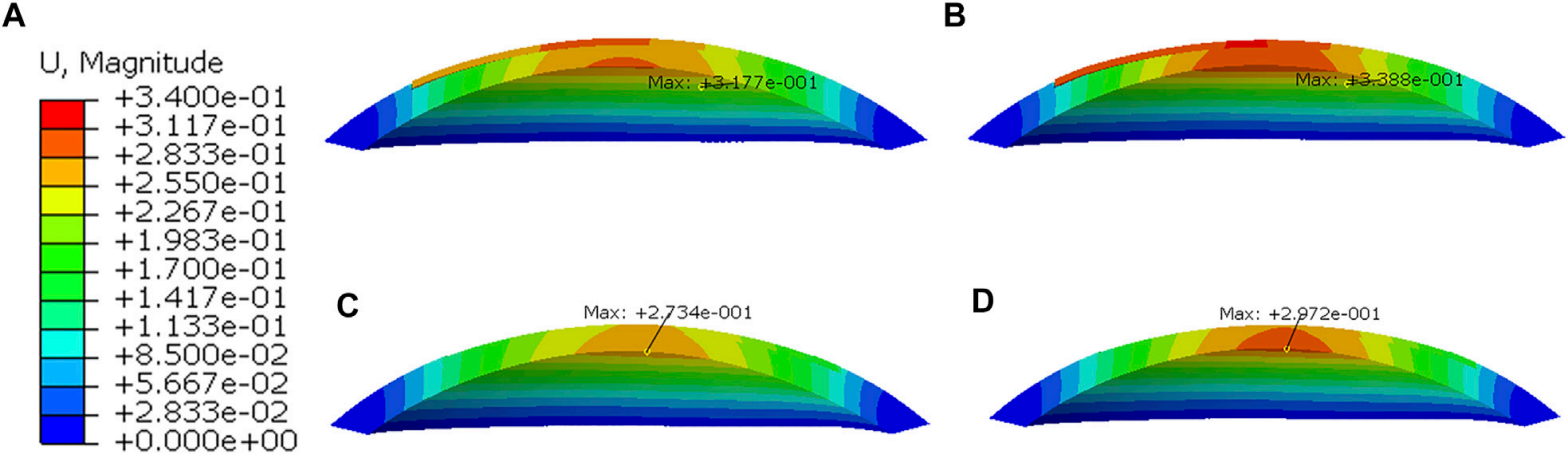
Displacement distributions after applying IOP of 19 mmHg. **(A)** Zero-pressure LASIK with -3D correction, **(B)** Zero-pressure LASIK with -6D correction, **(C)** Zero-pressure SMILE with -3D correction, **(D)** Zero-pressure SMILE with -6D correction.

Displacement distributions through a cross-sectional view of the cornea are presented in Figure 8. In all simulations, the greater the myopic correction was, the greater was the displacement induced by the applied IOP. Furthermore, the displacement distributions were the difference between LASIK and SMILE (both zero-pressure and image-based models). In the LASIK model, the displacement was concentrated at the corneal flap. However, the displacement distributions in the SMILE model are nearly symmetrical. The location of the maximum displacement in the SMILE model was at the center of the corneal cap. Overall, for the same myopic correction, the value of displacement in the LASIK model was higher than that of the SMILE model.

## Discussion

Laser refractive surgery, through ablation of the stromal tissue and the curvature of the corneal anterior surface, induced changes in the corneal refractive power. LASIK and SMILE have been proven to treat myopia safely and effectively with high satisfaction (Shen et al., 2016). A better understanding of the biomechanical behaviors of the LASIK and SMILE procedure may provide valuable references for the clinician. In this study, the finite element method was used to evaluate the influence of LASIK and SMILE surgery on the corneal displacement and Von Mises stress.

In the present study, the geometrical model of the cornea was reconstructed based on data measurements from the Oculus Pentacam. Then, the LASIK and SMILE refractive surgery were simulated based on a personalized model. However, the finite element corneal model incorporated the initial pre-stress, which is due to physiological IOP. An iterative algorithm was applied to retrieve the zero-pressure configuration of the intact cornea, LASIK and SMILE model. The results showed that the initial corneal pre-stress stiffens the response of the cornea, both in the intact cornea and under refractive surgery. The displacements along the *x-* and *y*-axes were higher obtained with the zero-pressure model than that of the image-based model. Furthermore, results for the zero-pressure SMILE model showed higher Von Mises stress on the corneal cap; however, the maximum stress was still less than that of the image-based SMILE model.

The mechanical properties of the human cornea are closely related to the integrity of the structure. The creation of a corneal flap/cap has an impact on the biomechanical behavior of the cornea. In the LASIK refractive surgery, a corneal flap is created by mechanical microkeratomes or femtosecond laser (Sugar, 2002), only hinge on a small part. However, during the SMILE procedure, a small incision is performed, which is located superiorly or superotemporally. In the present study, the flap hinge in the LASIK model and small incision in the SMILE model were also taken into account. The results showed that the displacement of the corneal flap was obviously higher than that of the corneal cap. Randleman et al. also demonstrated that the corneal postoperative strength after SMILE would be greater than that of LASIK due to the intact of the anterior lamellae (Randleman et al., 2008). Lenticule creation and removal in the SMILE procedure were assumed to provide a greater corneal structural stability and ocular surface integrity, and thus the biomechanical strength (Santhiago and Wilson, 2012). SMILE refractive surgery, which is far less invasive than LASIK, with a reduced incidence of postoperative corneal complications (Shah et al., 2011). Moreover, for both LASIK and SMILE, the displacement on the anterior corneal surface was different along the *x-* and *y*-axes, which may be due to the location of the corneal incision. However, the displacement along the *x-* and *y*-axes on the posterior corneal surface was similar. It indicated that the position of the corneal incision has relatively little effect on the posterior corneal surface compared to the anterior corneal surface.

It is generally believed that a certain residual stromal bed thickness is required after ablation to prevent ectasia (Reinstein et al., 2013). Refractive surgery with different ablation depths would influence the thickness of the residual bed under the same flap/cap. Under the same pressure, displacement values measured in LASIK (both zero-pressure and image-based model) with -6D correction were much larger than the ones that were measured in the model with -3D correction. Similar results could be found in the SMILE model. More displacement may increase the risk of corneal ectasia. In addition, the Von Mises stress measured in the LASIK and SMILE model with -3D correction was smaller than those with -6D correction. For example, when the IOP was 19 mmHg, the maximum corneal vertex stress in the zero-pressure SMILE model was 0.031 MPa in a -6D correction, while it was 0.028 MPa in a -3D correction, an increase of 10.71%. The higher Von Mises stress may trigger corneal damage, especially the microstructural tissues of the cornea. Katzengold et al. also demonstrated that the thicker the residual stromal bed was, the lower was the stress occurring as a result of the same IOP (Katzengold et al., 2021).

Furthermore, the creation of a corneal flap/cap affects the stress values on the corneal anterior and posterior surface. In LASIK, the flap is largely decoupled from the anchoring boundary of the corneal stroma, and hence has little effect as a mechanical component of the cornea (Schmack et al., 2005). Consistently, results showed that the stress on the flap was lower than that of the corneal cap. In contrast, LASIK increased the stress on the posterior corneal surface compared with the SMILE model. It should be noted that when comparing the maximum stress value between the corneal cap and residual stromal bed, the value on the cap was lower. This can be attributed to the fact that the corneal cap in the SMILE model was almost intact, which can carry the load together with the residual stromal bed. The increase in myopic correction resulted in greater stress on the cornea in both models.

With the same refractive surgery and myopic correction, the displacement and Von Mises stress of the residual stromal bed vertex increased linearly with the IOP. Fang et al. also presented similar findings (Fang et al., 2020). The results indicated that the IOP had a direct influence on the residual stromal bed. This might be an important reason for the instability postoperative outcomes after the refractive surgery. In this study, the simulation also showed that refractive surgery can affect displacement distributions. In the LASIK model, displacement distributions were not symmetric, while the displacement in the SMILE model was symmetric. It may be beneficial to consider creating a corneal cap in the refractive surgery to provide a more complete structure of the cornea.

There were several limitations in this study. For comparison purposes, the anisotropic, hyperelastic material properties were adopted in this study. However, the properties of the cornea were complex in physiological situations. Anisotropic viscoelastic material properties should be considered in further study. Moreover, although the geometrical model of the cornea was constructed based on the measured data, the perturbation of the collagen fibers was not considered. The corneal model could be improved by adding randomly dispersed collagen fibers. Finally, the finite element results were not compared with real LASIK and SMILE surgical outcomes, and postoperative results related to flap/cap were not measured. In the future, postoperative outcomes related to the LASIK and SMILE surgeries will be investigated, and the biomechanical behavior of the refractive surgery will be further elaborated.

## Conclusion

The present study evaluated biomechanical behaviors of the cornea after the LASIK and SMILE refractive surgery through a finite element method. The initial pre-stress of the cornea may stiffen the corneal response; therefore, the zero-pressure state of the cornea could not be ignored when considering the mechanical properties of refractive surgery. The corneal flap/cap could obviously change the biomechanical behaviors of the cornea. In this study, displacements of the SMILE model were obviously lower than those of the LASIK model under the same IOP. Moreover, the corneal cap may bear more load compared with the corneal flap. However, the maximum stress in the SMILE model was still smaller than that of the LASIK model. Overall, this study indicates that SMILE refractive surgery has advantages over the LASIK procedure in the aspect of corneal biomechanical stability.

## Data Availability

The original contributions presented in the study are included in the article/Supplementary Material. Further inquiries can be directed to the corresponding authors.
